# Lessons on applying a trauma‐informed research approach in health professions education scholarship

**DOI:** 10.1111/medu.15745

**Published:** 2025-06-13

**Authors:** Helen Anne Nolan

**Affiliations:** ^1^ Warwick Medical School University of Warwick, Gibbet Hill Coventry England UK

## Abstract

**Background:**

Health professions education (HPE) research routinely explores trauma‐related subjects, yet trauma impacts may remain overlooked. Trauma‐informed approaches and guiding principles provide a framework for interactions with those affected by trauma and advocate for accommodation of trauma impacts to promote recovery and inclusion. My own reflections on experiences of trauma‐related research in medical education led to consideration of how trauma‐informed approaches may be applied in qualitative research. This paper identifies and discusses strategies that may be implemented in HPE research.

**Approach:**

The paper begins by exploring the rationale for trauma‐informed approaches and how these relate to contemporary HPE research. Examples of emergent approaches from published literature in various disciplines, and how these can accommodate the needs of groups and individuals impacted by trauma and promote inclusion to enhance research outcomes, are identified and discussed. Approaches are situated in key aspects of the research process.

**Findings:**

Trauma‐informed research has been recently adopted in various disciplines and may act as an effective adjunct to established ethics protocols, although no published accounts in HPE research were identified. Application of trauma‐informed approaches and reflexivity in research design may pre‐emptively identify trauma‐related needs. Trauma‐informed approaches can be applied to create safety, trust and empowerment during data collection interactions. Measures to manage participant distress are discussed.

Principles for community engagement and participation and their role in in HPE research are considered, exploring how these overcome trauma impacts (e.g. by sharing power and supporting inclusion), and enhance research outputs.

Implications of trauma for researcher wellbeing and research culture are also acknowledged, and trauma‐informed approaches may systematically support these needs. Approaches to research dissemination should acknowledge existing oppressive structures and consider alternatives, in partnership with affected communities.

**Conclusions:**

This paper considers an emergent area of research practice, highlighting strategies that are currently under‐explored and may enhance HPE research by addressing trauma impacts, and supporting stakeholder collaboration in research. Approaches may be readily implemented and adapted to the research context. Adoption of these approaches should be supported by trauma‐informed research training to enhance research culture, and institutions more widely.

## Abstract 2

Arising from reflections on research relating to trauma in medical education, this paper draws on the literature to consider how trauma‐informed approaches may be applied in HPE research.

## 1 INTRODUCTION

### 1.1 Health professions education research

Contemporary health professions education (HPE) research engages with an array of subjects associated with experiences of trauma, or deemed to be ‘sensitive’^1^, ^2^ i.e. topics associated with risk of moral, emotional or mental distress.^3^ Topics widely recognised as belonging to these categories include but are not limited to, life‐limiting conditions and dying, mental health and trauma, domestic and sexual violence, abuse, discrimination, inequities, racism and conflict.^3^, ^4^, ^5^ The potential for emotional impacts should not, however, deter scholarly exploration of such subjects.^4^ HPE researchers look to shed light on the unexplored, advancing knowledge and driving change.^6^ In many circumstances trauma‐related research participation is tolerated well^7^, ^8^ or identified as cathartic. Sharing of participants' stories may enable reinterpretation of individuals' experiences in the wider social or structural context and harness benefits for others.^9^ Ensuring positive experiences of participation necessitates recognition of traumatising sources and structures,^10^ and associated impacts; areas still frequently overlooked in HPE research.^11^, ^12^ The potential for traumatic impacts should not be considered solely in relation to the topic,^13^ but should additionally be seen as being contextual, relational and dynamic^4^. Deeper recognition of the implications of trauma in HPE research may provide an avenue to enhance participant and researcher experience, and research outcomes.^14^ Trauma‐informed care, a framework to support recognition and accommodation of trauma impacts and promote recovery,^15^, ^16^ is increasingly implemented in healthcare and HPE.^11^, ^17^ More recently, this framework has been applied by qualitative researchers in various disciplines to promote wellbeing and inclusive, equitable research^10^, ^12^, ^14^, ^18^ My experience of undertaking trauma‐related qualitative research with medical educators prompted reflection on the need to embed trauma‐informed approaches in research, leading to exploration of established scholarly practice from alternative disciplines. Applications and perspectives from a range of disciplines and relevance to HPE research are considered here.

To evaluate the role of trauma‐informed approaches in research, it is first necessary to more thoroughly consider constructs of trauma and their relevance in HPE research. A commonly‐cited definition describes trauma as arising from ‘an event …..or set of circumstances that is experienced as physically or emotionally harmful or life‐threatening and has lasting adverse effects on an individual's functioning and mental, physical, social, emotional, or spiritual well‐being’,^15^ with this definition encompassing additional entities beyond those reflected in diagnostic criteria for post‐traumatic stress disorder.^19^ Exposure to trauma and associated impacts are highly prevalent. Using more restrictive criteria, a WHO World Mental Health Survey found that 70% of participants reported experiencing lifetime trauma.^20^ Complex traumatic impacts from sustained exposure to prolonged, often interpersonal, traumatic events, may result in more severe responses.^21^ Racial, cultural and historical traumas arise and recur due to prevailing hierarchies and discrimination at micro, meso and macro levels.^9^, ^14^ This definition depicts how power differentials are frequently intrinsic to traumatic experiences and underscore attendant disruption to feelings of trust and security,^21^ conditions essential for creating rapport and meaningful exchange in the research process.^22^

Failure to recognise how trauma arises and affects individuals may sustain power differentials and traumatising practices during research.^12^, ^23^ In many circumstances, the precise nature of participants' trauma histories will not be known to researchers. Stakeholders in HPE research include educators, learners and patients, with the latter categories more liable to experiencing unfavourable power differentials.^24^, ^25^, ^26^, ^27^ Patients in many settings (e.g. psychiatry, women's health) are likely to have encountered trauma.^24^ Medical learners encounter various risks of (re)traumatisation during learning and training^28^, ^29^ and which may be revisited during research. Trauma risks may only be recognised by researchers ‘in real time’ when confronted unexpectedly with participants' trauma narratives or reactions.^1^, ^30^ Failure to consider trauma‐related needs may sustain barriers to research participation for affected individuals,^23^ resulting in their needs being de‐prioritised in research agendas, sustaining social and health inequities.^23^ Irrespective of the topic, it is worth considering that participants^14^, ^23^, ^31^ ‐ and researchers^32^, ^33^ ‐ may have experiences of trauma, noting its widespread prevalence.

Requirements for institutional ethics approval remain the mainstay of safeguarding against risks to participants.^8^, ^12^, ^14^, ^23^, ^34^, ^35^ These are founded on principles of ‘respect for autonomy’, ‘beneficence’, ‘non‐maleficence’ and ‘justice’ and emphasise consideration of risk, benefit and consent.^14^, ^34^ Ethical approaches applied in HPE research emanate from those established in scientific disciplines.^27^, ^34^, ^36^ Prevailing approaches in biomedical and quantitative traditions reflect a positivist stance, suggesting that all risks can be measured and balanced.^27^, ^34^ Qualitative approaches are commonly employed in HPE research to explore meanings derived from experience.^10^ These are characterised by attention to context^34^ and the need to iterate ‐ hallmarks of rigour and quality in this research tradition.^37^ Responsiveness and reflexivity result in actions that cannot be anticipated and documented in ethics protocols.^12^, ^14^, ^34^ Rigid, pre‐emptive, even ‘paternalistic’^34^ ethics stipulations may lack the nuance and sensitivity required in qualitative approaches.^12^, ^23^, ^38^ Efforts to protect ‘vulnerable’ participants may result in their further disempowerment, a particular challenge where participatory approaches are intended.^34^, ^38^, ^39^ Scholars recommend that ideas of ‘vulnerability’ be replaced instead by ‘susceptibility to harm’, as this explicitly recognises the contextual nature of this risk and illuminates ways in which it may be actively mitigated.^40^

Requirements of ethics protocols tend to focus predominantly, if not exclusively, on the needs of participants.^3^, ^4^, ^14^, ^41^, ^42^, ^43^ Although safeguarding of participant wellbeing is ethically and morally necessary, researchers' own wellbeing may be comparatively neglected^5^, ^34^, ^44^ – risks now substantiated in several fields.^4^, ^5^, ^32^, ^42^, ^44^, ^45^, ^46^ Academic institutions where research occurs, and their established practices and norms, may also pose risks of researcher traumatisation.^43^ These factors highlight the need for careful attention to trauma in research ethics, design and delivery.^14^ Applying a system‐wide, trauma‐informed research approach also attends to staff safety and wellbeing.^14^, ^47^

### 1.2 Trauma‐informed approaches

**FIGURE 1:**
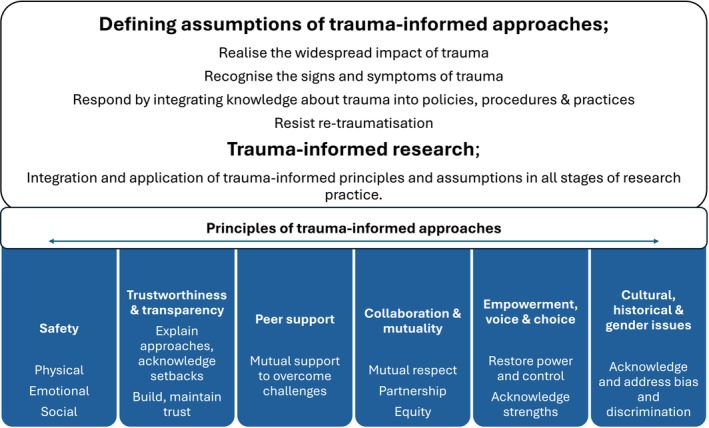
Assumptions and principles of trauma‐informed approaches. [Color figure can be viewed at wileyonlinelibrary.com]

In response to the expanding evidence base and recognition of trauma prevalence and impacts, trauma‐informed approaches (also termed trauma‐informed care or practice) have emerged as a strengths‐based, person‐centred framework to support recognition and accommodation of trauma impacts.^15^, ^16^ Trauma‐informed approaches are established in diverse settings e.g. healthcare, social care, education, justice systems^48^ and are intended to operate at all levels of the social ecology, from individual practice to organisational and systems practice and policy.^16^ Core assumptions and principles of trauma‐informed approaches are described (Figure 1). Overarching motivations include minimising risks of (re)traumatisation to stakeholders, and promoting resilience and recovery. The first precondition is awareness of trauma evidence, including its prevalence, associated impacts, features suggestive of trauma responses and measures to reduce risks of (re)traumatisation. Beyond circumstances where individuals' trauma histories are known, or direct exploration of trauma‐related topics, trauma‐informed approaches are intended to be applied in all interactions and processes as ‘universal precautions’,^31^ acknowledging trauma's often hidden nature.^14^, ^31^ As evidence regarding trauma impacts^49^ and its association with minoritised experiences and inequities expands,^8^, ^50^ so too does the application of TI approaches in research to promote participant empowerment and inclusion^12^, ^24^, ^25^ and mitigate risks of traumatisation to both participants and researchers.^12^, ^23^, ^51^ Described as both praxis and philosophy,^12^ trauma‐informed research recognises the impact of trauma and integrates trauma‐informed principles and assumptions in all stages of research practice, prioritising safety and empowerment.^14^ Researchers in various disciplines have created guidance and frameworks for trauma‐informed research e.g., mental health, social work, as complementary to existing ethics procedures.^8^, ^12^, ^14^, ^18^, ^23^, ^30^, ^52^

Despite the necessary focus on issues of inequities, social justice and various other trauma‐related topics in HPE research,^1^ and research traditions that may be associated with traumatisation,^53^ there currently appears to be limited attention to trauma‐related concerns in this research field. In undertaking a trauma‐related qualitative interview study with unfamiliar medical educators, I necessarily reflected on the alignment between research planning and ethics protocols, and real‐world experiences of discussing these issues with professional acquaintances. Arising from these experiences and reflections, I wished to explore how trauma‐informed practice could be applied and integrated in HPE research, and strategies to enact this. Emergent approaches from a range of disciplines are described here and their relevance to HPE research considered.

## 2 APPROACH

Having considered the prevalence and sources of trauma, including structural and systemic sources, this discursive paper synthesises evidence relating to rationale for trauma‐informed research and its relevance to HPE research. Although it is beyond the scope of this paper to provide a systematic overview of literature relating to all aspects of trauma in research, this paper highlights emergent accounts and approaches to trauma‐informed research from various disciplines. Discussion particularly consider interview and focus group studies, although strategies may also apply in other research contexts. Additionally, it considers afresh established research practices that align to trauma‐informed research, with the overarching aim of identifying practical strategies that may be implemented in HPE research.

To initially explore if and how application of trauma‐informed approaches in research is described, general searches were conducted in Google scholar, identifying published papers,^12^, ^23^ practical guidance^47^, ^54^ and academic webpages.^55^ To assess for other key papers, basic title and keywords searches for “trauma‐informed research”, “trauma‐informed research practice” and “trauma‐informed qualitative research” were conducted in five databases (Medline, PsycINFO, Scopus, CINAHL and Web of Science). Sources were reviewed and subsequent citation chaining (i.e. searching out sources cited in papers and reviewing sources citing the identified papers) was undertaken, as this is identified as an efficient strategy in searching disparate evidence.^56^ Sixty‐five unique sources were identified of which 30 papers were identified as most relevant, as they described application of or provided recommendations for trauma‐informed approaches in research. Papers were relatively recent, starting in 2020 and came from disciplines including e.g., healthcare, social care and public health,^9^, ^14^, ^18^, ^23^, ^24^, ^25^, ^30^, ^41^, ^57^, ^58^, ^59^, ^60^, ^61^ sociology and social work,^8^, ^10^, ^12^, ^31^, ^38^, ^39^, ^47^, ^54^, ^62^, ^63^ and education^51^, ^64^ psychology^52^, ^65^ and law.^66^ Notably none from HPE research were identified. Additional evidence relating to key strategies recommended for trauma‐informed research design and delivery e.g. reflexivity,^5^, ^67^ professional boundaries,^68^ researcher wellbeing^4^, ^32^, ^33^, ^43^, ^45^, ^69^ are also discussed with reference to HPE research. The paper is structured in relation to key aspects of the research process from planning and data collection through to dissemination and impact, and ongoing researcher wellbeing, and considers approaches to enhance trauma‐informed HPE research. Approaches are not intended to replace or supersede, but instead complement, existing ethics frameworks.^55^

### 2.1 Reflexivity

I am a cisgender white woman working as a healthcare educator and researcher. My interest here arises from wider interest and curriculum development in trauma‐informed medical education, which encourages exploration of trauma and associated health outcomes across medical curricula, and attends to trauma impacts on patients, learners and clinicians.^70^ I am interested in understanding how trauma‐informed approaches apply in further areas of my professional practice including research. I adopt the previously cited definition of trauma that acknowledges individual's interpretation of events as significant in determining whether they are experienced as traumatic and recognises trauma as arising from structural and systemic issues and power structures.^15^ As such, I recognise trauma‐informed approaches as a systems‐wide approach and aspect of inclusive practice.

## 3 UNDERTAKING TRAUMA‐INFORMED RESEARCH

### 3.1 Research planning

A fundamental requirement for trauma‐informed research is foundational knowledge regarding the recognition of risks and impacts, and the role of self‐care,^8^, ^12^, ^25^, ^42^, ^45^ with training identified as essential for effective implementation.^18^, ^58^ Considering trauma's prevalence and potential relevance to many topics in HPE research, these key issues should be explored in HPE qualitative researcher training. Research design and planning should confirm justification of the research rationale and likely benefit, particularly for those under study and impacted by trauma.^14^ Researchers should carefully reflect on how trauma may be relevant to participants through exploration and reflection on their historical, sociopolitical and cultural context, ideally through direct preparatory interaction with participant communities to inform design (Section 3.3).^8^ This familiarisation at the outset goes hand‐in‐hand with reflexivity around researcher's own positionality, including social location and experiences including own trauma. A prerequisite for authentic research participation and sharing of experiences is psychological safety.^14^ Conditions to create this should be considered in research design and planning, and reflexively throughout delivery, particularly in relation to data collection interactions.

### 3.2 The research interaction; data collection

Commonly employed qualitative data collection strategies include one‐to‐one interviews and focus groups to derive meaning from experiences.^10^ The ‘inherently interpersonal’^12^ nature of interactions in research interviews and focus groups means that researcher‐participant interactions, or between focus group participants themselves, may be impacted by larger systems of oppression, with the risk of replicating power hierarchies for those affected by trauma.^10^, ^12^ Trauma may disrupt personal narrative.^57^ Key steps to sense‐making post‐trauma include speaking about, reordering and taking control of the experience and reestablishing social connection.^21^ Research participation represents an opportunity for discussion of these experiences, which may be the participant's first disclosure.^61^ Despite careful attention to research conduct, role boundaries may become blurred^68^ and the interaction risks becoming reoriented as a therapeutic one,^5^, ^23^ issues considered in Section 3.4.1. Various measures can accommodate trauma impacts, empower effective discussion and maintain positive relationships with participants. These factors, alongside increased researcher confidence in engaging with participants, may enhance data quality.^46^, ^59^

#### 3.2.1 Preparing for the interaction

Information sharing and consent should be ongoing and active processes,^9^, ^30^, ^60^ facilitated by timely, transparent sharing of study information in accessible language,^41^ and opportunities ensure clarity and understanding between researcher and participant.^8^, ^12^ The ability to assert one's rights and preferences may be affected for trauma survivors, so advance sharing of question areas and communicating the option to omit question(s) at the participant's discretion^54^ may support participants to establish boundaries regarding what they wish to share.^12^ Some researchers recommend measured sharing of own experiences or reason for interest in the topic, as a mechanism to level hierarchies and build rapport.^41^, ^42^, ^54^, ^65^, ^68^ Researchers should pre‐emptively consider approaches here and identify how appropriate boundaries will be maintained (Section 3.4.1).^9^, ^54^, ^61^, ^68^

Organisation of the interaction should, where feasible, consider participant needs and preferences relating to location, format, timing, recording arrangements and presence of a peer supporter to engender trauma‐informed principles of safety, trust and choice in the process.^9^, ^12^, ^38^, ^54^ Online settings are increasingly utilised. Researchers have considered the relative benefits and drawbacks of this format for interviewing^66^ and focus groups^30^ in trauma‐related circumstances. They highlight detailed considerations to enhance feasibility and identify areas for further evaluation (e.g. accessibility).^66^ Online settings should be managed by advance testing of technology, ensuring familiarity with its use and providing clear instructions for participants.^54^

Advance planning and (co‐created) safety protocols for participant distress or retraumatisation^13^, ^63^ should consider resourcing, ensuring sufficient time for breaks or presence of an additional facilitator or peer supporter to assist distressed participants.^12^, ^54^ Information regarding support resources should be identified by the researcher in advance, rather than having to locate these reactively when distress arises, to ensure predictability and control.^8^, ^12^, ^14^, ^54^ Resources should be relevant to participants' location and needs.^30^ Incentives for participation, and how these are provided, should be considered as they may be perceived by trauma survivors as coercive when offered in the context of unaddressed power hierarchies (Section 3.3).^12^

#### 3.2.2 During the interaction

Psychological safety can be maintained by setting the scene and attending to the physical environment, revisiting the purpose of the research, providing further opportunities for participant clarification and highlighting options to decline questions and control the pace of the interaction.^12^, ^14^, ^18^, ^30^, ^54^, ^66^ Participant access to the question list during interactions may enhance predictability and safety.^8^ Where intending to explore trauma‐related issues, it is recommended to commence with a neutral question that is conducive to sharing and rapport‐building.^12^ Longman et al. describe using a “hill” analogy, encouraging participants to “look down” on their experiences to enable more dispassionate recounting, rather than reliving, of traumatic experiences.^18^ Clarity at the outset regarding the protocol for managing distress and participants' agency here may promote trust and empowerment. In online settings, options to contact the (co‐)facilitator directly ensures participant safety and privacy.^54^ A key aspect of trauma awareness is recognition of signs of acute (re)traumatisation; commonly recognised as a ‘fight‐flight‐freeze’ response which may include appearing blank, distracted or overwhelmed, agitated or angry.^9^, ^12^ Simple grounding techniques or measures to refocus are recommended to support emotional regulation,^12^, ^14^ although use has not been evaluated. Informing participants of how distress is being managed can provide reassurance in a group situation. When offering participants options, these should be clear, discrete choices that do not cause further overwhelm. Resuming with a resilience‐based question can also support refocusing e.g. ‘What/who has enabled you to cope with these experiences?’.^12^

#### 3.2.3 Closing the interaction

Sensitive closure and debrief, with assessment of wellbeing,^54^ and focus on resilience, can support wellbeing after the interaction.^12^ Signposting to previously‐identified wellbeing resources as a matter of course can normalise the need to access these.^57^ With participants' prior agreement, brief follow‐up in the subsequent days may be appropriate to identify those who require further supports.^12^ In addition to summarising and providing an opportunity for questions or clarifications, transparency regarding the next steps, use of data and inviting participant feedback on the process are recommended.^14^, ^54^ This commitment to collaboration not only enhances the immediate experience but may support participants' continued engagement or future co‐production.^10^, ^59^

### 3.3 Community engagement

Consideration of trauma‐informed principles of collaboration and empowerment can enhance research by creating meaningful partnerships, assigning value to participant priorities and sharing power. These approaches additionally acknowledge participants' strengths and resilience.^10^, ^30^, ^31^, ^38^, ^39^, ^58^, ^59^ A spectrum of approaches to participant collaboration and empowerment in research are advocated including co‐production, patient/public engagement, community participatory action research and peer research. These overarching aims and approaches are discussed here under the term “community engagement”. Rationale includes improving quality, acceptability and transparency of research process and outputs, by ensuring better alignment to community priorities.^10^, ^24^, ^59^, ^71^ Community engagement and participatory research also challenge hierarchies and give voice to minoritised or oppressed groups whose needs may be overlooked,^38^, ^39^, ^72^ motivations all clearly congruent with trauma‐informed principles of empowerment, choice and overcoming cultural, historical and gender issues.^15^ Studies from e.g., education, social science, health research, and with groups impacted by trauma (e.g. abuse survivors, migrants) describe community engagement and participation as both conducive to trauma‐informed research and underpinned by trauma‐informed principles.^8^, ^10^, ^59^ Collaborative engagement with community organisations may lend credibility to researchers and support recruitment by enabling access to participants and support resources via a trusted organisation.^9^, ^10^, ^18^, ^30^, ^58^, ^59^ These considerations are relevant to HPE research where stakeholders may be impacted by trauma and are liable to experiencing unfavourable power differentials.^24^, ^25^, ^27^ Although community (e.g., patient, student) perspectives are regularly implied in HPE research, these voices remain underutilised in determining research processes.^26^, ^73^ Insights from trauma‐informed community engagement in other contexts may enhance HPE research.

The precise engagement approach taken will be influenced by research aims and context,^65^ mechanisms available for engaging communities (e.g. within education/healthcare organisations, via community organisations or previous participants) and resource availability.^14^ Trauma‐informed studies describe establishing community advisory groups to co‐develop research protocols and advise throughout delivery.^12^, ^59^, ^65^ Hargrove et al. reflect on forming a team faculty‐student participatory trauma‐informed research team to explore effects on trauma on learning, placing emphasis on parity for student experience and perspectives in addition to academic perspectives.^64^ Elsewhere, community researchers (individuals already embedded in the community), were employed to deliver various aspects of trauma‐informed research projects.^18^, ^58^, ^62^ Commonly identified, unifying measures to enable effective, trauma‐informed community engagement include research teams undertaking initial rapport‐building with communities to intentionally generate trust which may have been disrupted by traumatic experiences.^12^, ^38^, ^59^ Commencement should also include co‐learning and mutual upskilling regarding trauma and its potential relevance to the community, acknowledgement of resilience and cultural considerations.^24^, ^25^, ^38^, ^39^ These insights should inform collectively‐agreed research objectives and policies to guide the delivery of each project stage.^24^, ^59^ Project agreements should provide predictability and support participation, while maintaining flexibility to accommodate evolving needs.^12^, ^38^ Intentional efforts to recognise and legitimate unique experiences and skillsets can create more equitable conditions for collaboration, task allocation and enable all research contributions to be valued.^24^, ^25^, ^38^, ^39^ Reflexivity can support identification and critical awareness of assumptions and interpretations held by the research team (Section 3.4.3).^24^, ^72^, ^74^ Beyond notional or symbolic value, compensation for contributions of research partners should be comparable to those of other ‘academic’ team members^12^, ^24^, ^75^. Fraley et al. summarise guiding frameworks and considerations for compensation.^59^

Despite signalled intentions regarding parity and partnership, effective co‐production is not guaranteed.^59^, ^71^ Lonbay et al. (2021) discussed experiences of co‐production in mental health research. Connotations of mental health difficulties and othering language risked reinforcing barriers between ‘patient’ versus ‘researcher’.^39^ Shankley et al. discuss (2023) the dilemma of empowering while also protecting potentially traumatised research partners in undertaking participatory research, particularly when confronted by adverse research findings.^38^ Here partners were informed of continuous optionality and supported to make their own choice regarding participation at various project stages. Trauma‐informed research partnerships should demonstrate accountability and transparently acknowledge setbacks, and be realistic regarding potential benefits and limitations of research outputs.^10^, ^12^, ^62^, ^72^ Similarly, acknowledging limitations to parity and differing positionality and privilege between team members is necessary to preserve trust and to empower research partners.^24^, ^39^ Intentionality and reflexivity are required to understand and overcome pitfalls, factors particularly relevant when engaging those affected by trauma.^23^, ^24^ Where full, authentic co‐production is not possible, measures ensuring some collaboration should be attempted, to gain insight into community experiences and priorities. This may include consultation to inform researchers' understanding of trauma in a particular community.^38^ Literature review, engagement with media resources and implementation of participant feedback may also achieve this aim.^12^, ^18^, ^65^ Beyond the delivery of a discrete project, community engagement and collaboration may also sustain partnerships,^59^ support community capacity building and creation direct research impact for and with affected communities.^10^, ^12^, ^24^

### 3.4 Researcher wellbeing

Wellbeing risk in qualitative research is often evaluated in relation to perceived ‘sensitivity’ of subjects, and usually in relation to risks to the participant. This approach downplays potential risks to researchers themselves^3^, ^4^, ^43^, ^69^ and which are now increasingly recognised.^4^, ^5^, ^32^, ^42^, ^44^, ^45^, ^46^ Training and formalised wellbeing support, even amongst ‘professional’ researchers, are reported as lacking,^4^, ^41^, ^43^, ^76^ and as overlooking risks of emotional harm.^3^, ^31^, ^46^ Where wellbeing measures are instituted, these are commonly self‐initiated by researchers and reactively, after a significant event.^4^ Healthcare educators' route to research may be organic, as a practitioner‐researcher, and not necessarily associated with formalised researcher training,^77^ leaving them ill‐equipped for recognising and managing risks, particularly relating to own wellbeing. Although codes of practice operate with respect to clinical roles to safeguard profession wellbeing, these do not encompass research activity.^68^

#### 3.4.1 Professional boundaries

Qualitative approaches including interview and focus group studies rely on rapport‐building and interpersonal connection via mutual sharing and researcher self‐disclosure, described by researchers as “levelling the playing field” and embodying empathy with participants.^68^ This however risks blurring the researcher's protective professional boundary^68^ and challenges role clarity.^9^, ^23^ HPE research is commonly undertaken by clinicians whose primary role and professional identity is care‐giving and therapeutic.^14^, ^33^, ^42^ Inevitably, research and therapeutic interviewing draw upon common skills.^68^ Participants may experience the interaction as therapeutic, unbeknownst to researchers and despite their distinct intentions. Unintentional therapeutic effects may be beneficial to participants. However, this “therapeutic payoff” may also raise unexpected issues for participants, and which researchers are ill‐equipped to manage, thereby additionally compromising both parties' wellbeing.^68^ Approaches such as debrief are regularly suggested, yet researchers may not feel skilled in this practice.^68^, ^69^ Despite incidental participant benefits, research is not an appropriate substitute for trauma therapy, nor should researchers assume this role.^51^, ^68^

#### 3.4.2 Researcher trauma

Researchers' engagement with particular subjects may be influenced by own personal trauma histories,^3^, ^5^, ^24^, ^41^ and a desire to ameliorate circumstances for others similarly affected, by influencing knowledge and policy.^3^ Researchers may participate as an ‘insider researcher’, identifying as belonging to the group of interest, highlighting the artificial dichotomy between ‘researcher’ and ‘participant’ status.^24^, ^25^ In circumstances where researchers feel unable to declare this shared identity this may impede access to appropriate support.^41^, ^61^ Vicarious trauma, i.e. cumulative cognitive change in beliefs and thinking resulting from chronic empathic engagement with trauma‐related issues,^78^, ^79^ may arise not only from sustained engagement with oppressed groups or trauma survivors, but also from the iterative and immersive acts of data collection, transcription and analysis of distressing content.^5^, ^42^, ^57^, ^69^, ^80^ Recognition of these issues and needs (e.g. wellbeing risks, blurring of boundaries) is recommended from the outset of research planning and can be partly addressed through (self)‐reflexivity.

#### 3.4.3 (Self)‐reflexivity for researcher wellbeing

Although used primarily as a mechanism to promote objectivity, credibility and rigour in analysis ‘by examination of the lens through which the researcher views the phenomenon’,^67^ reflexivity is a commonly‐cited strategy for researcher wellbeing. Self‐reflexivity effectively enables researchers to consider how their own background and positionality impact research and how the research process impacts them,^24^, ^81^ and the need for self‐care.^82^ Reflexivity facilitates transparency and accountability for research interpretations and conclusions by considering power differentials between researcher and participant.^14^ Although researcher recognition and engagement with own vulnerability may be perceived as compromising research objectivity and rigour, it is imperative that researchers also recognise their own responses to trauma‐related issues to preserve research quality, and own and participant wellbeing.^14^, ^41^, ^83^ This aligns with the idea of ‘trauma stewardship’, which refers to proactively recognising and addressing the impacts of secondary trauma.^84^

Despite an ‘overabundance of concern’ in the literature regarding reflexivity in sensitive qualitative research, limited evidence of training is noted, leaving many researchers ill‐equipped to undertake this vital practice.^4^ Although undertheorised,^5^ one commonly‐used approach to researcher self‐reflexivity is journalling,^4^, ^5^, ^42^ content of which may be private or discussed in supervision or peer dialogue. Reflective practice is habitually undertaken by healthcare professionals to support professional development.^85^ Although distinct processes, these skills may enable researchers to practice reflexivity.^14^ Formal exploration of reflexivity in researcher training, ensuring early inception in practice, is recommended.

#### 3.4.4 Wellbeing in context

Other factors influencing wellbeing include interpersonal relationships (peers, supervisors, research team), disciplinary culture and organisational context and ethos.^3^, ^41^, ^43^, ^45^, ^51^ Trauma‐informed approaches are intended to operate across individual, interpersonal (e.g. supervisory) and organisational levels and can be applied accordingly to research contexts. Trauma‐informed approaches emphasise the role of relational and peer support in reducing and overcoming trauma‐impacts.^15^ Although junior researchers or students may benefit from regular supervision, quality varies and supervision may become more scarce post‐training.^51^ Trauma‐informed research settings should ensure consistent access to peer or supervisory support, wellbeing checks and debrief after emotionally intense research activity and ongoing mentorship,^9^, ^34^ alongside informal social contact time for researchers.^45^, ^69^ Debrief and peer support should also be extended to research partners in the case of co‐produced or participatory research.^39^

Wellbeing is also context‐bound by a wider supportive organisational culture.^43^ Resource allocation and funding bids should ideally cost wellbeing needs, including supervised debrief, manageable work schedules (e.g. spacing between intense interviews)^3^, ^9^, ^14^, ^38^ and access to mental health support for researchers.^4^, ^9^ Where supervision or specialist trauma support is not available within the organisation, external support should be accessed.^18^, ^68^ Funders^69^ and ethics committees^43^, ^55^ should encourage these practices to support culture change. Positive research leadership can enable the creation of these conditions.^69^ Leadership that embodies trauma‐informed approaches may support the creation of healthy and effective environments, and instil these approaches in emergent researchers.^4^, ^76^ Just as hierarchies and inherent power structures should be acknowledged in participant interactions, consideration of wider institutional and academic hierarchies and practices that perpetuate traumatising conditions is required.^86^, ^87^

### 3.5 Dissemination and impact

Working with oppressed or traumatised groups can be associated with researcher feelings of powerlessness, guilt and frustration, particularly where research impact is lacking.^3^, ^46^ Evidence‐policy gaps, with failure to translate evidence to drive social or health improvements, are well described, including in relation to trauma impacts.^88^ Overarching objectives relating to social justice, equity and trauma recovery, should inform approaches to research dissemination to harness benefits for affected individuals and communities. Approaches for the use of data and dissemination should be undertaken with regard for principles of participant safety and choice, alongside informed consent and privacy.^52^ Findings should be shared directly with participants and their communities,^14^ with opportunities for debriefing and discussion of the next steps. Researcher partners themselves can take an active role in communicating findings with their wider community,^65^ ensuring transparency and impact. Approaches to translation of evidence should also incorporate collaboration; affecting change *with* communities,^12^, ^30^ alongside communication to and lobbying healthcare and education policymakers and leaders for socially just transformation. Consideration of novel routes and platforms e.g. accessible media, podcasts,^30^, ^65^ beyond those established in academic settings and structures that replicate systems of power and privilege e.g. peer‐reviewed journals^86^, ^89^ and that recognise participants stories and efforts^18^ is recommended. Open science advocates for the sharing of data with the wider research community allowing new questions to be explored while using existing data to minimise risks of participant retraumatisation. Meticulous care for consent and anonymity will be required in this emergent field.^52^

## 4 CONCLUSION AND IMPLICATIONS FOR PRACTICE

Trauma may often be relevant to research, by virtue of the topic under consideration, as well as and the research context and practices, and wider systems of power that operate, including in HPE research. Trauma‐informed research accommodates issues intrinsic to HPE including wellbeing, equity and power‐sharing through approaches that are currently under‐utilised in this field. Adopting a contemporary definition that locates trauma experiences within individual, sociocultural and historical contexts,^64^ and informed by a review of the emerging and growing body of literature in this area, this paper provides a comprehensive overview of the ways in which trauma may be relevant, and reenacted *or* ameliorated throughout the research process. Considerations and practical strategies that can be adapted to context and applied in undertaking trauma‐informed HPE research from design and data collection through to dissemination and impact are summarised in Table 1.

**TABLE 1:** Summary of strategies and considerations for undertaking trauma‐informed research.

Research activity	Trauma‐informed strategy or consideration	Comments
Research planning	Address training needs regarding trauma and trauma‐informed approaches Reflexively consider relevance of trauma and power to study topic, and participants' wider context and researcher's own positionality Evaluate rationale and likely benefit of research for groups affected by trauma Consider how to collaborate with participant community e.g. via community organisations, participatory research to inform research agenda, protocols and delivery	Understanding of trauma sources, prevalence and impacts is essential for undertaking informed research
Data collection interaction (Interviews, focus groups)	**Before the interaction** Provide clear, accessible participant information in advance Address participant questions, confirm consent and manage these as ongoing processes Address participants needs and preferences for the interaction (e.g. location, format, accessibility needs) Develop safety protocol to manage distress Access resources required for participant safety, choice and empowerment e.g. additional facilitator, peer supporter, time needed to pause, wellbeing resources Test technology, including with participants, for online interactions Consider if/how incentives will be offered, avoiding any sense of coercion **During the interaction** Address questions, confirm consent and highlight confidentiality Highlight option for participant control and choice; to pause or discontinue, omit questions, safety protocol, Share question list to share control with participants Open with a resilience‐focused question Be vigilant for signs of distress In the event of distress, offer, clear simple options **Closing the interaction** Address participant questions, clarifications, acknowledge participant efforts and resilience Enquire regarding wellbeing and signpost to wellbeing resources/supports Invite participant feedback on the process Discuss next steps including how data will be used, planned project outputs and whether/how participant wishes to be updated or remain involved. Discuss option to “check‐in” with participant(s) in the coming days to confirm wellbeing Reflect on own wellbeing and safety, and access support, debrief or practice self‐care	Trauma may disrupt personal narrative, ability to assert rights and preferences Researcher‐participant interactions, or between focus groups participants may be impacted by larger systems of oppression, with risk of replicating power hierarchies for those affected by trauma
Community engagement	Engagement approach will be influenced by research aims, context and resource availability. **General principles include:** Undertake rapport‐building and co‐learning with community regarding trauma and resilience Co‐create research objectives and policies, while remaining flexible to evolving needs Recognise unique experience and skillsets Compensate research partners Transparently acknowledge setbacks, limitations of research and collaboration Consider how to sustain community engagement in implementing research outputs and build community capacity	Collaboration and partnership are advocated with those whose needs and contributions may be overlooked due to abuse, power differentials and structures
Researcher wellbeing	**Topic** **Researcher practice** Reflect on own possible responses to the research topic Consider how participant rapport will be managed and how personal experience will be shared Develop self‐reflexivity practice e.g. reflexive journalling, reflexive dialogue, to recognise own responses to trauma‐related issues **Research context** Ensure researchers have access to formal and informal wellbeing and peer support Consider how institutional, disciplinary culture and leadership demonstrate and prioritise trauma awareness Institutions, funders should promote trauma‐informed training and prioritise researcher wellbeing	Wellbeing risks for researchers should be considered in relation to the topic, in addition to personal and professional context
Dissemination and impact	Dissemination should harness benefits for affected individuals and communities. Findings should be shared with and implemented directly by participants and their communities, including via research partners/communities Consider alternative, accessible routes for dissemination to overcome established power systems of power and privilege e.g. in academia	Impact should be harnessed to justify trauma‐related research and promote recovery

Strategies and considerations are overlapping and apply across different research activities. For example, research wellbeing should be considered through all study stages. Approaches are not tightly mapped to particular trauma‐informed principles, as these may align to numerous principles.

Application of some trauma‐informed approaches may be recognised as ‘basic good practice’.^14^ However, existing approaches can be deployed with greater intentionality and awareness, specifically of trauma impacts and their significance in research. Approaches and strategies have not been tightly mapped to particular trauma‐informed principles, as these may align to numerous principles and researcher should reflect on how these apply and serve needs in their own research context. The approaches are overlapping and complementary, e.g. effective research dissemination may promote researcher fulfilment and wellbeing.^43^ Considerations identified are aligned to multi‐level nature of trauma‐informed approaches, applying to individual, interpersonal (e.g. supervisory) and institutional practices. Applying trauma‐informed approaches in research, as complementary to research ethics,^18^, ^55^ may exceed minimum expectations to “do no harm”,^8^ by supporting participant resilience and recovery and improving HPE research outcomes. Trauma awareness and trauma‐informed training are identified as a crucial precondition for enabling trauma‐informed research at individual, interpersonal and organisational research level,^18^ and for driving research cultural shifts. Richer understanding of trauma acknowledges its relevance beyond research topics and recognises structures and hierarchies that affect research practice and experiences. This may be particularly relevant in HPE contexts where biomedical definitions of trauma still prevail.^90^ These issues should be considered in HPE researcher training, particularly in relation to qualitative approaches, in learners' formative research experiences, and within communities of scholarly practice. As trauma‐informed research advances, approaches and participant community experiences should be evaluated to develop approaches and optimise benefits.

## CONFLICT OF INTEREST STATEMENT

None.

## AUTHOR CONTRIBUTIONS

**Helen Anne Nolan:** Conceptualization; investigation; writing – original draft; methodology; visualization; writing – review and editing; project administration.

## ETHICS STATEMENT

Ethical approval was not required.

## Data Availability

Data sharing not applicable to this article as no datasets were generated or analysed during the current study.
